# The impact of smoking on the clinical outcome of locoregionally advanced nasopharyngeal carcinoma after chemoradiotherapy

**DOI:** 10.1186/s13014-014-0246-y

**Published:** 2014-11-26

**Authors:** Shan-Shan Guo, Pei-Yu Huang, Qiu-Yan Chen, Huai Liu, Lin-Quan Tang, Lu Zhang, Li-Ting Liu, Ka-Jia Cao, Ling Guo, Hao-Yuan Mo, Xiang Guo, Ming-Huang Hong, Hai-Qiang Mai

**Affiliations:** Sun Yat-sen University Cancer Center, State Key Laboratory of Oncology in South China, Collaborative Innovation Center for Cancer Medicine, Guangzhou, P. R. China; Department of Nasopharyngeal Carcinoma, Sun Yat-sen University Cancer Center, 651 Dongfeng Road East, Guangzhou, 510060 P. R. China; GCP Center, Sun Yat-sen University Cancer Center, Guangzhou, P. R. China

**Keywords:** Nasopharyngeal carcinoma, Prognostic factor, Radiotherapy, Smoking

## Abstract

**Background:**

Cigarette smoking is a common risk factor for developing nasopharyngeal carcinoma. However, the relationship between smoking and clinical outcomes remains uncertain.

**Methods:**

The patients who participated in this study were drawn from a randomized clinical trial, for which the purpose was to compare the efficacy of induction chemotherapy plus concurrent chemoradiotherapy with that of induction chemotherapy plus radiotherapy in patients with locoregionally advanced nasopharyngeal carcinoma. The patients who ever smoked were divided into the following categories of cumulative smoking exposure based on the duration of smoking and the quantity of cigarettes smoked: light, short-term smokers; light, long-term smokers; heavy, short-term smokers; and heavy, long-term smokers. A log-rank test and Cox models were used to assess the association between smoking and the clinical outcomes of overall survival (OS), failure-free survival (FFS), locoregional recurrence failure-free survival (LRFFS) and distant failure-free survival (DFFS).

**Results:**

We found that ever-smokers experienced significantly shorter LRFFS times than never-smokers (5-year LRFFS rates: 85.8% vs. 88.5%, P = 0.022). The amount of smoking was significantly associated with FFS (P = 0.046) and LRFFS (P = 0.001) in the different ever-smoker groups. The amount of smoking was associated with LRFFS [P = 0.002, HR = 2.069 (95% confident interval (CI), 1.298-3.299)] even after a multivariable adjustment.

**Conclusions:**

Smoking increases the risk of locoregional recurrence. Furthermore, the amount of smoking influences the prognosis of smokers, and these effects are dose-dependent.

## Background

Nasopharyngeal carcinoma (NPC) is rare in most parts of the world but is endemic in southern China and southern Asia. There were approximately 84,400 incident cases of NPC and 51,600 NPC-related deaths in 2008 throughout the world [1]. A variety of risk factors have been correlated with NPC, including alcohol, tobacco, Cantonese-style salted fish, occupational exposures and herbal drugs [2]. Among these, smoking has been definitively associated with the risk for the onset of NPC because former and current smokers display increased risks compared to never-smokers [3]. It was reported that 301 million smokers lived in China in 2010 [4]. To date, little information exists regarding cigarette smoking and the progression and outcome of NPC, which reveals the importance of analyzing the prognostic role of cigarette smoking among NPC patients.

We hypothesized that cigarette smoking is associated with the biological prognosis and clinical outcome for NPC. To verify our hypothesis, we used the data from a large, prospective, randomized clinical trial that compared the efficacy of induction chemotherapy plus concurrent chemoradiotherapy (IC+CCRT) with that of induction chemotherapy plus radiotherapy (IC+RT) in patients with locoregionally advanced nasopharyngeal carcinoma [5,6].

## Methods

### Ethics statement

The clinical trial and this retrospective study were approved by the Research Ethics Committee of Sun Yat-sen University Cancer Center, Guangzhou, PR China. All patients had signed informed consent forms in the clinical trial, and informed consent was given by participants for their clinical records to be used in this study. Patient records were anonymized and de-identified prior to analysis.

### Patient selection

The patients who participated in this study were drawn from a randomized clinical trial, for which the purpose was to compare the efficacy of induction chemotherapy plus concurrent chemoradiotherapy (IC+CCRT) with that of induction chemotherapy plus radiotherapy (IC+RT) in patients with locoregionally advanced nasopharyngeal carcinoma; the clinical trial was conducted from February 2002 to April 2005 in our institute. All these patients had been treated with a unified conventional, 2-dimensional technique in line with the treatment policy for NPC at Sun Yat-sen University Cancer Center. Every patient had been given conditional radiotherapy at 2 Gy per fraction and 5 daily fractions per week, with a total dose of 68-70Gy. In the IC+CCRT group, the patients were treated with 2 cycles of floxuridine +carboplatin (FuDR, 750 mg/m^2^, d1-5; carboplatin, area under the curve, AUC=6). Radiotherapy had been administered to these patients one week after completing chemotherapy. Carboplatin (AUC=6) had been given to the patients in this group on days 7, 28 and 49 while undergoing treatment with RT. Two cycles of FuDR d1-d5+carboplatin (FuDR, 750 mg/m^2^, d1-5; carboplatin,area under the curve, AUC=6) were administered to the patients in the IC+RT group. All of these patients had received RT one week after completing chemotherapy. Only 7 patients had interruption of RT. The details of the therapeutic process could be seen in the previous studies regarding the clinical trial [5,6].

In the present study, the variables that were available for analysis included a history of ever smoking (ever-smokers or never-smokers), the amount of smoking presented as cigarettes per day (CPD) (never smoked, ≤9, 10-19, 20-29, ≥30), the duration of the smoking interval (never smoked, <10,10-19.9, 20-29.9, 30-39.9, ≥40 yr) and time since the cessation of smoking (≥10, 5-9.9, ≤4.9 yr). These criteria were chosen based on previous studies [7-13]. All of the above data regarding smoking were prospectively collected before diagnosis. At the time of diagnosis, we divided all the NPC patients through self-reporting into ever-smokers and never-smokers, which were defined as having smoked 100 cigarettes or less than 100 cigarettes in the lifetime according to Centers for Disease Control and Prevention (CDC) of America, respectively [14]. Ever-smokers included former smokers and current smokers, who were defined as having quit smoking for more than 1 year or who consistently smoked within the last year, respectively. We further categorized the patients who ever smoked into the following groups of cumulative smoking exposure based on the duration of smoking and the quantity of cigarettes smoked: light, short-term smokers (≤19 CPD for ≤19.9 yr); light, long-term smokers (≤19 CPD for ≥20 yr); heavy, short-term smokers (≥20 CPD for ≤19.9 yr); and heavy, long-term smokers (≥20 CPD for ≥20 yr). Cut-off for cumulative smoking exposure was selected based on the midpoint of the four categories of duration of smoking and quantity smoked. Cutoff values of VCA IgA and EA IgA were set at 1:80 for VCA IgA and 1:10 for EA IgA, which were based on previous studies [15,16]. Acute mucosal toxicity were evaluated and recorded in accord with Common Terminology Criteria for Adverse Events (CTCAE) version 2.0.

### Statistical analysis

All the events were measured from the date of random assignment, and the statistical tests were performed using the SPSS17.0 software (SPSS Inc., Chicago, IL). Descriptive statistics were calculated for patient and disease characteristics according to smoking. The associations between the categorical variables were assessed using the ϰ^2^ test. The survival rates were calculated using the Kaplan–Meier method, and the differences between the groups were compared using the log-rank test. Cox regression models were used to calculate the HRs with the associated 95% CIs for the associations between clinical outcomes and the following multiple smoking parameters: history of ever smoking (ever-smokers or never-smokers), the amount of smoking (never smoked, ≤9, 10-19, 20-29, ≥30 yr), the duration of the smoking interval (never smoked, <10,10-19.9, 20-29.9, 30-39.9, ≥40 yr), the time since smoking cessation (≥10, 5-9.9, ≤4.9) and the cumulative smoking exposure (light, short-term smokers; light, long-term smokers; heavy, short-term smokers; heavy, long-term smokers). The Cox regression models were adjusted for the effects of age, gender, T stage, N stage and treatment arm. Two-sided P values less than 0.05 were considered statistically significant.

## Results

### Baseline characteristics

As previously described by Huang [5,6], no significant differences were observed in FFS, locoregional control or distant control between the IC+RT and IC+CCRT groups. The IC+CCRT regimen did not improve the overall survival or failure-free survival of patients with locoregional advanced nasopharyngeal carcinoma compared with the IC+RT regimen [5,6].

In the present study, the mean age of all the patients was 43 yr (range: 18-65 yr). The characteristics of the study population according to smoking status are described in Table 1. Of the 400 patients, 207 (51.7%) were never-smokers and 193 (48.3%) were ever-smokers. The ever-smokers included 34 (17.6%) former smokers and 159 (82.4%) current smokers. The numbers of ever-smokers in each of the cumulative smoking exposure groups were as follows: 32 (16.8%) patients were light, short-term smokers; 18 (8.4%) were light, long-term smokers; 57 (29.8%) were heavy, short-term smokers; and 86 (45.0%) were heavy, long-term smokers. Age, the tumor size (T1-2, T3-4), the lymph node status (N0-1, N2-3), the clinical stage, the treatment arm, RT doses, RT interruptions, CT courses, acute mucosal toxicity(1-2,3-4), VCA-IgA (Positive(≥1:80), Negative(<1:80)), EA-IgA (Positive(≥1:10), Negative(<1:10)) and the median follow-up time did not differ between the never-smokers and ever-smokers, but there was a difference regarding gender (P<0.001) (Table 1). Age, the tumor size (T1-2, T3-4), the lymph node status (N0-1, N2-3), the clinical stage, the treatment arm, RT doses, RT interruptions, CT courses, acute mucosal toxicity(1-2,3-4), VCA-IgA (Positive(≥1:80), Negative(<1:80)), EA-IgA (Positive(≥1:10), Negative(<1:10)) and the median follow-up time were not different between the 4 cumulative smoking groups, but there was a difference regarding gender (P=0.005)(Table 2).

**Table 1:** **Baseline characteristics of 400 patients with locoregionally advanced nasopharyngeal carcinoma**

	**Ever 193(48.3%)**	**Never 207(51.8%)**	**P value**
**Age(yr), median(range)**	45(18-63)	41(18-65)	0.969
**Gender**			<0.001
**Female**	3(1.6)	84(40.6)	
**Male**	190(98.4)	123(59.4)	
**T stage**			
**1-2**	29(15.0)	28(13.5)	0.668
**3-4**	164(85.0)	179(86.5)	
**N stage**			0.541
**0-1**	92(47.7)	105(50.7)	
**2-3**	101(52.3)	102(49.3)	
**Clinical stage**			0.222
**3**	103(53.4)	123(59.4)	
**4**	90(46.6)	84(40.6)	
**Treatment arm**			0.617
**IC + CCRT**	99(51.3)	101(48.8)	
**IC + RT**	94(48.7)	106(51.2)	
**RT dose(Gy),median(range)**	72(68-78)	72(68-78)	0.773
**CT course, median(range)**	3(1-5)	3(1-5)	0.079
**RT interruption**			0.936
**yes**	4(2.1)	3(1.4)	
**no**	189(97.9)	204(98.6)	
**AMT**			0.559
**1-2**	182(93.8)	196(95.1)	
**3-4**	11(6.2)	10(4.9)	
**VCA-IgA**			0.833
**Positive(≥1:80)**	174(90.2)	187(96.6)	
**Negative(<1:80)**	19(9.8)	19(3.4)	
**EA-IgA**			0.181
**Positive(≥1:10)**	151(78.2)	172(83.6)	
**Negative(<1:10)**	42(21.8)	34(16.4)	
**Median follow-up yr, median(range)**	6.5(0.2-9.0)	6.9(0.6-9.3)	0.236

*Abbreviations:* IC + CCRT = induction chemotherapy plus concurrent chemoradiotherapy and IC + RT = induction chemotherapy plus radiotherapy. RT = radiotherapy. CT = chemotherapy. AMT = Acute mucosal toxicity.

**Table 2:** **Baseline characteristics of 193 ever-smokers with locoregionally advanced nasopharyngeal carcinoma**

	**Light, short-term**	**Light, long-term**	**Heavy, short-term**	**Heavy, long-term**	***P*** **value**
	32(16.8%)	18(8.4%)	57(29.8%)	86(45.0%)	
**Age, yr, median**	40(20-58)	50(36-60)	39(24-59)	49(10-66)	0.452
**Gender**					0.005
**Female**	1(3.1)	1(12.5)	0	0	
**Male**	31(96.9)	17(87.5)	57(100)	86(100)	
**T stage**					0.368
**1-2**	7(21.9)	1(5.6)	8(14.0)	13(15.1)	
**3-4**	25(78.1)	17(94.4)	49(86.0)	73(84.9)	
**N stage**					0.463
**0-1**	16(48.5)	6(33.3)	25(43.9)	45(52.3)	
**2-3**	17(51.5)	12(66.7)	32(56.1)	41(47.7)	
**Clinical stage**					0.189
**3**	15(46.9)	14(75.0)	34(59.6)	43(50.0)	
**4**	17(53.1)	4(25.0)	23(40.4)	43(50.0)	
**Treatment arm**					0.641
**IC + CCRT**	16(50.0)	11(61.1)	32(56.1)	41(47.7)	
**IC + RT**	16(50.0)	7(38.9)	25(43.9)	45(52.3)	
**RT dose(Gy),median(range)**	72(68-78)	72(68-78)	72(68-78)	72(68-78)	0.118
**CT course, median(range)**	2(2-5)	32(1-5)	22(1-5)	22(1-5)	0.470
**RT interruption**					
**Yes**	2(6.2)	0(0)	1(1.8)	1(1.2)	0.337
**No**	30(93.8)	18(100)	55(98.2)	85(98.8)	
**AMT**					0.731
**1-2**	30(93.8)	18(100)	52(92.9)	80(93.0)	
**3-4**	2(6.2)	0(0)	4(7.1)	6(7.0)	
**VCA-IgA**					0.330
**Positive(≥1:80)**	30(93.8)	17(94.4)	47(83.9)	79(91.9)	
**Negative**	2(6.3)	1(5.6)	9(16.1)	7(8.1)	
**EA-IgA**					0.121
**Positive(≥1:10)**	27(84.4)	17(94.4)	39(69.6)	67(77.9)	
**Negative**	5(15.6)	1(5.6)	17(30.4)	19(22.1)	
**Median follow-up yr (range)**	6.8(0.5-8.7)	6.9(1.1-8.3)	6.5(0.2-8.6)	6.2(0.6-9.0)	0.572

*Abbreviations:* IC + CCRT = induction chemotherapy plus concurrent chemoradiotherapy and IC + RT = induction chemotherapy plus radiotherapy. AMT = Acute mucosal toxicity.

### Survival results according to cumulative smoking exposure and multivariate analysis for different endpoints

The median follow-up time was 80.2 mo (range=40.9-92.1 mo). During the follow-up, 149 patients (37%) died, 121 patients (30%) had distant metastasis, and 62 (16%) patients experienced a locoregional recurrence.

**Figure 1:**
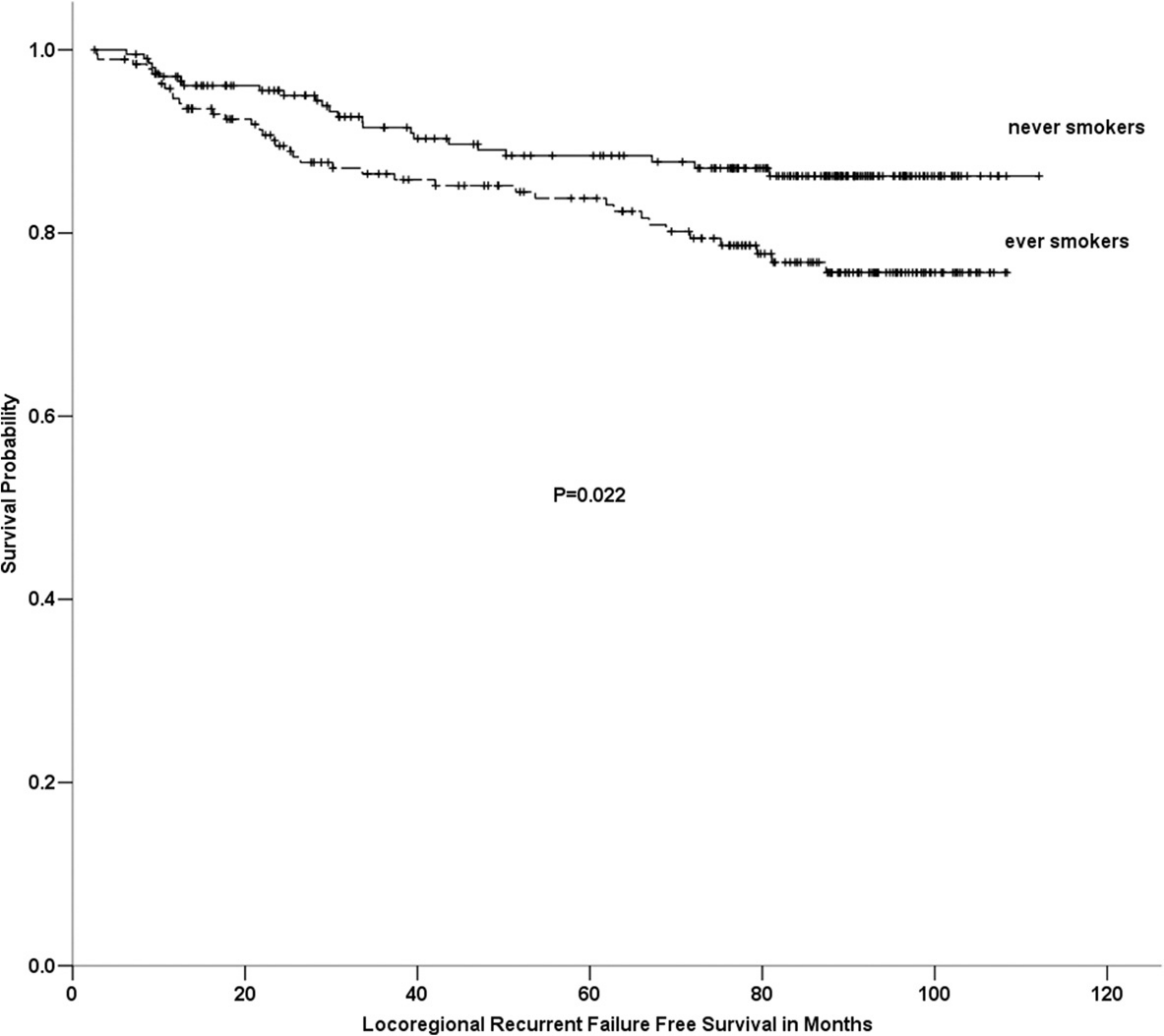
**Comparison among ever-smokers and never-smokers on locoregional recurrence failure free survival of 400 patients with locoregionally advanced nasopharyngeal carcinoma.**

For the entire group, the actuarial 5-yr OS, FFS, LRFFS and DFFS rates were 71.5%, 62.6%, 86.2% and 71.5%, respectively. OS, FFS and DFFS were not associated with smoking between the ever-smokers and never-smokers. As shown in Figure 1, the 5-yr LRFFS rates in ever-smokers and never-smokers were 85.8% and 88.5% (P=0.022), respectively. After adjusting for age, gender, the T stage, the N stage, the clinical stage and the treatment arm, smoking was still related to LRFFS [P=0.002, HR=2.223 (95% CI, 1.351-3.658)] (Table 3).

**Table 3:** **Multivariable analysis of LRFFS among all patients using a Cox regression model**

**Characteristics**	**HR**	**95%CI**	***P*** **value**
**Age**	1.002	0.969-1.035	0.924
**Gender**	0.134	0.016-1.149	0.067
**T stage**	0.871	0.426-1.781	0.705
**N stage**	0.915	0.569-1.473	0.715
**Clinical stage**	1.799	0.705-4.589	0.219
**Treatment arm**	1.160	0.605-2.224	0.654
**Smoking amount**	2.223	1.351-3.658	0.002

*Abbreviations:* HR = hazard ratio; CI = confidence interval.

**Figure 2:**
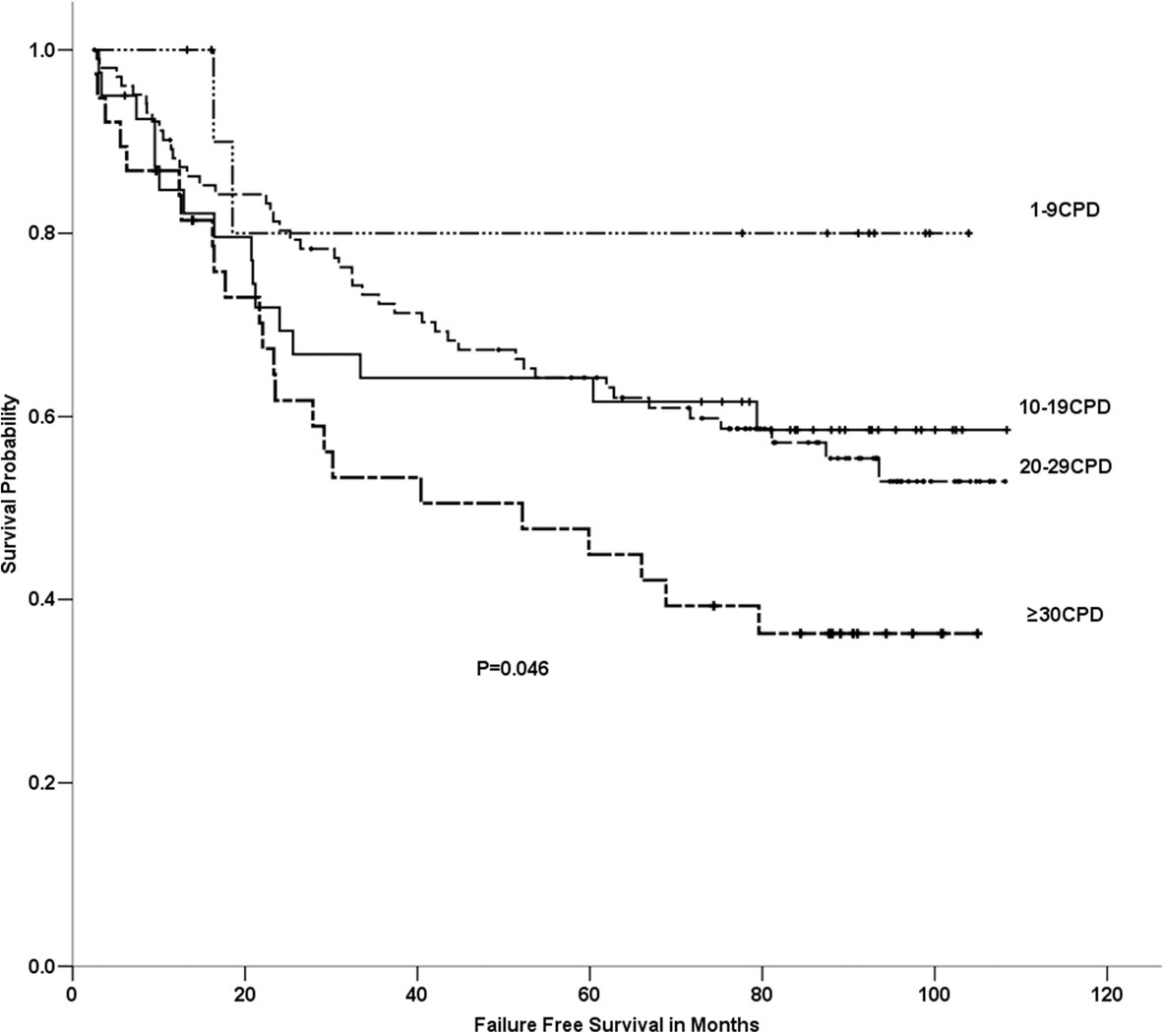
**Comparison among different amount of smoking groups on failure free survival of 400 patients with locoregionally advanced nasopharyngeal carcinoma.**

**Figure 3:**
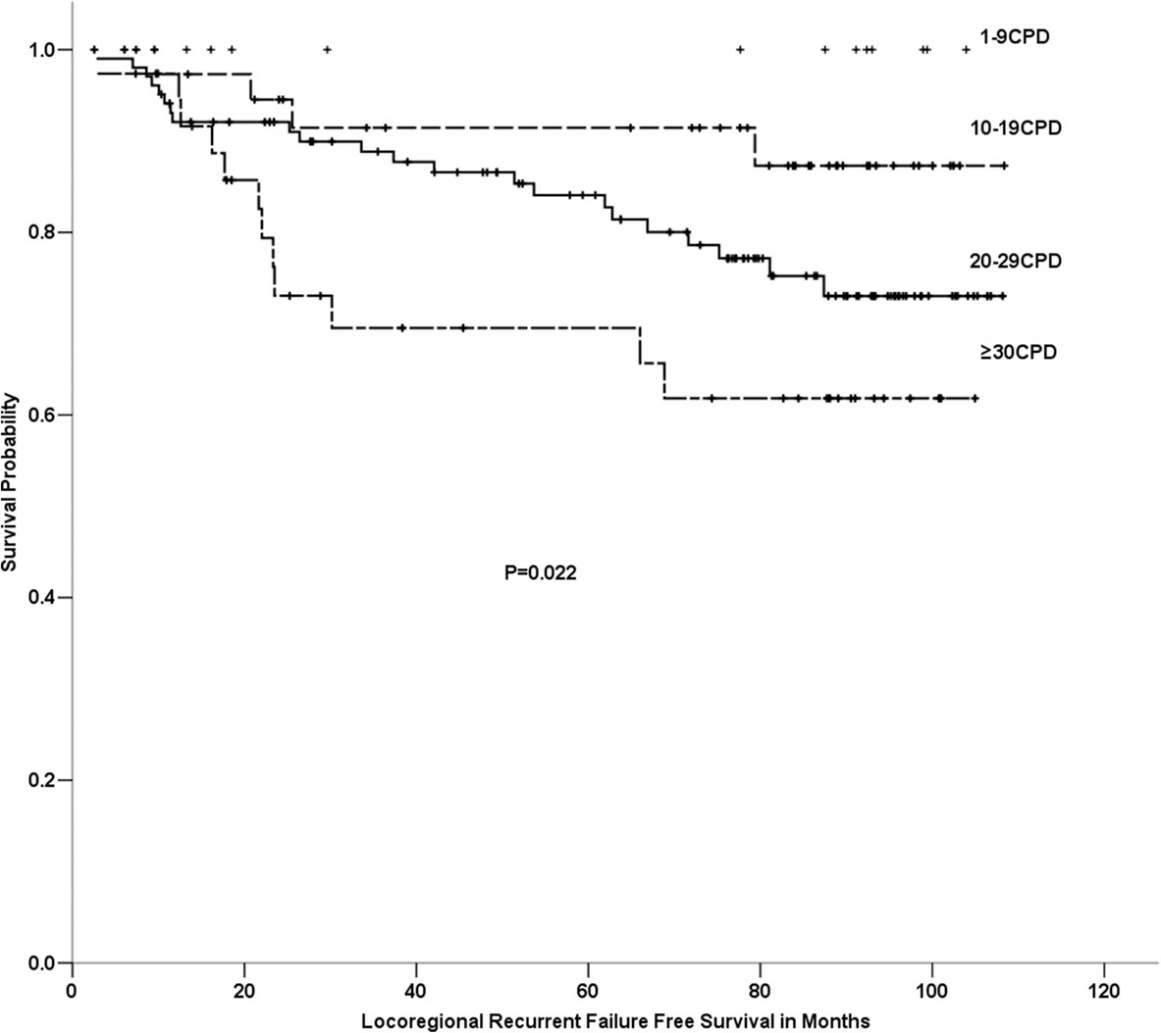
**Comparison among different amount of smoking groups on locoregional recurrence failure free survival of 400 patients with locoregionally advanced nasopharyngeal carcinoma.**

No significant difference in the OS, FFS, LRFFS or DFFS rates between the former and current smokers was evident. The 5-yr OS rates in the light, short-term smokers, the light, long-term smokers, the heavy, short-term smokers and the heavy, long-term smokers were 62.0%, 77.8%, 74.3% and 63.0%, respectively. No significant difference was found among these 4 cumulative smoking groups in OS, FFS, DFFS or LRFFS. We found that the amount of smoking was significantly associated with FFS (P=0.046) and LRFFS (P=0.022) in the ever-smokers, but this factor was not associated with OS or DFFS (Figures 2 and 3). The duration of smoking was not associated with OS, FFS, DFFS or LRFFS. After adjusting for age, gender, the T stage, the N stage, the clinical stage and the treatment arm, the amount of smoking was significantly associated with LRFFS [P=0.002, HR=2.233 (95% CI, 1.351-3.658)].

## Discussion

Tobacco use, which is responsible for approximately one-third of all cancer deaths annually, is the largest preventable cause of cancer and cancer mortality [17]. Nearly half of our study cases were current or former smokers, which emphasizes the fact that smoking is significantly associated with the risk for the onset of NPC. Jia et al. reported that individuals who reported a high cumulative amount of smoking had an increased risk of NPC compared with never-smokers [18]. A number of studies have reported that smoking is a predictor of unfavorable survival among head and neck cancer patients [17,19-22], and most of these studies have shown that smoking before treatment is associated with a worse clinical outcome. A multicenter European study demonstrated that cigarette smoking adversely affects survival, in particular, in cases of a tumor originating at the endolarynx [20]. A prospective cohort study indicated that smoking status was the strongest predictor of survival in both current smokers [hazard ratio (HR) =2.4; 95% CI, 1.3-4.4] and former smokers (HR =2.0; 95% CI, 1.2-3.5), and the data revealed a significant association with poor survival among patients with head and neck squamous carcinoma [21]. Chen et al. has reported that, among male NPC patients, smokers have a significantly reduced 5-yr overall survival and locoregional recurrence free survival compared with non-smokers [23]. Ouyang et al. found that the risks of death, progression, locoregional relapse, and distant metastasis were significantly higher for former and current smokers than never smokers [24]. Both of the two recent researches were retrospective studies.

The present study utilized data from a randomized clinical trial that included 400 patients with locoregionally advanced carcinoma. We prospectively collected data of smoking before diagnosis, so the results of our study may be more credible than the two retrospective studies [23,24]. We have found that cigarette smoking is significantly associated with an increased incidence of locoregional disease failure. However, we did not find the relevance between cigarette smoking and OS, FFS and DFFS in the present study. Because some of the patients who experienced local regional recurrence could be cured by salvage therapy. Novel treatment techniques and strategies-including precision radiotherapy, endoscopic surgery, radical neck dissections third-generation chemotherapy regimens, and targeted therapies and immunotherapy have provided hope for patients with recurrent nasopharyngeal carcinoma [25,26]. So smoking does not have an impact on OS. Smoking is a factor for tumor growth, and cigarette smoke acts as a mutagen and DNA damaging agent that drives tumor initiation in normal epithelial cells [27,28]. Cigarettes could cause genetic mutations and methylation, thereby resulting in the transformation of epithelial cells in the nasopharynx, an area in contact with potentially carcinogenic substances in cigarettes directly through inhalation [29]. So cigarette smoking has impact on local epithelial cells directly, although not having sufficient power to significantly influence disease distant metastasis.

Smoking is a factor for tumor growth, and cigarette smoke acts as a mutagen and DNA damaging agent that drives tumor initiation in normal epithelial cells [27,28]. Cigarettes could cause genetic mutations and methylation, thereby resulting in the transformation of epithelial cells in the nasopharynx, an area in contact with potentially carcinogenic substances in cigarettes directly through inhalation [29]. DNA mutations can influence local resistance to radiation and chemotherapy [30] but should increase also distant metastasis that is frequently causes of death. However the present study showed the 5-year distant failure free survival (DFFS) in ever-smokers and never-smokers was 70.4% and 70.7%, respectively (P>0.05). Because the effect of chemotherapy in lowering distant failure is controversial in head and neck cancer, including NPC. Adjuvant cisplatin and fluorouracil chemotherapy did not significantly improve DFFS after concurrent chemoradiotherapy in locoregionally advanced nasopharyngeal carcinoma [31,32]. Also there was no difference in DFFS between those patients treated with induction chemotherapy followed by chemoradiotherapy and those who received chemoradiotherapy alone [33,34]. Although DNA mutations have impact on resistance to chemotherapy, actually they have little impact on disease distant metastasis.

In our study, the patients who smoked more cigarettes per day were significantly associated with FFS and LRFFS. After adjusting for the T stage, the N stage, the clinical stage, age and gender, the amount of smoking was still an independent prognostic factor for LRFFS according to the Cox regression analysis. It reveals that the more cigarette smoking, the more risk of locaregional disease recurrence and disease progression in patients of nasopharyngeal carcinoma. This is in accordance with a previous study. Crosignani et al. reported that heavy tobacco smoking appeared to worsen the prognoses of male laryngeal cancer patients in a dose-dependent manner [19]. The exact explanation of this dose-dependent effect remains uncertain. It has been hypothesized that the more cigarette smoking, the more impact on the epithelial cells of nasopharynx. As a result, the risk of disease recurrent would be higher in those who smoked more cigarettes. We did not find any association between time duration of smoking and prognosis. It means that the time duration of smoking is not a prognostic factor for nasopharyngeal carcinoma. The actual mechanism about the relationship of the amount of smoking as well as time duration and prognosis would be searched by further molecular study. In the present study, former smokers did not differ from current smokers regarding LRFFS; the number of patients who had quit smoking may have been too small to reveal any differences.

In our study, acute mucosal toxicity was not different between ever-smokers and never-smokers. The relationship between acute mucosal toxicity and smoking of NPC patients remains uncertain. The result about acute mucosal toxicity is in accordance with several previous studies. Vatca et al. reported that no-smokers had a significant increase in the risk of developing severe mucositis in oropharyngeal squamous cell carcinoma [35,36]. Other studies yet did not find differences between smoking and mucositis in tumors including head and neck cancer [37-39]. Only one study regarding nasopharyngeal carcinoma found that smoking history are high risk factors of serious mucositis after radiotherapy for 102 NPC patients in 2005 [40]. But there were some limitations to the above study. The enrolled patients were treated with conventional radiotherapy, using cobalt 60 or X-ray. And the sample size was too small to provide valuable evidence. The association between smoking and acute mucosal toxicity need to be explored by further large-scale sample studies.

The exact mechanisms underlying the impact of smoking on cancer genesis and progression remain elusive. Recent studies showed that nicotine decreases the effectiveness of RT and chemoradiotherapy [30,41]. A previous publication revealed a relationship between smoking status and tumor oxygen unloading capacity [42]. Tobacco smoking is known to have immunosuppressive effects on local tissues via induction of pro-inflammatory cytokines and chemokines and suppression of antigen recognition and response. Cigarette smoking affects a wide range of immune functions impacting innate and adaptive host immunity, smoking induced changes in antibody production, particularly in response to foreign antigens that impinge on the respiratory mucosa [43]. Moreover, chronic exposure to cigarette smoke or nicotine causes T cell unresponsiveness, and nicotine-induced immunosuppression may result from its direct effects on lymphocytes [44,45]. Cigarette smokers exhibit impaired NK cytotoxic activity, increased pro-inflammatory cytokines production by peripheral mononuclear cells, and increased T-cell proliferative response to mitogens [46,47]. Smoking is a factor for tumor growth, and cigarette smoke acts as a mutagen and DNA damaging agent that drives tumor initiation in normal epithelial cells [27,28]. Cigarettes could cause genetic mutations and methylation, thereby resulting in the transformation of epithelial cells in the nasopharynx, an area in contact with potentially carcinogenic substances in cigarettes directly through inhalation [29]. Nicotine consistently reduced the cytotoxic effects of DNA-damaging agents used in treatment, such as cisplatin and UV and gamma radiation [30]. Besides, Epstein–Barr virus (EBV) also played a role in the progression of nasopharyngeal carcinoma. EBV reactivation in B cells could be triggered by cellular products of tumor cells [48]. Conversely, EBV might act as a promoter for tumorigenesis in a feedback loop. EBV reactivation is associated with the elevations of levels of cytokines and growth factors, ie, interleukin-6, interleukin-10, transforming growth factor-β1, and vascular endothelial growth factor, which could contribute to cell proliferation, immune system perturbation, and angiogenesis [49-52].

Our study has some limitations. First and foremost, other smoking forms were not recorded, such as hookah smoking, smoking a pipe, second-hand smoke and so on. In addition, our data were collected from a single cancer center, and it is not known whether the conclusion from our center can be expanded to other regions. Multicenter clinical trials may be able to verify the value of pretreatment smoking cessation in locoregional advanced nasopharyngeal carcinoma patients.

## Conclusions

We determined the effects of cigarette smoking on the clinical outcomes of NPC patients. Smoking leads to an increased risk of locoregional disease recurrence. Furthermore, the amount of smoking influences the prognosis of smokers, and patients who smoked more cigarettes per day have a greater risk of locoregional disease recurrence than those who smoke fewer cigarettes per day.

## Abbreviations

OS: Overall survival
FFS: Failure-free survival
LRFFS: Locoregional recurrence failure-free survival
DFFS: Distant failure-free survival
CI: Confident interval
NPC: Nasopharyngeal carcinoma
IC + CCRT: Induction chemotherapy plus concurrent chemoradiotherapy
IC + RT: Induction chemotherapy plus radiotherapy
FuDR: Floxuridine
AUC: Area under the curve
CPD: Cigarettes per day
yr: Year
CDC: Centers for disease control and prevention
CTCAE: Common terminology criteria for adverse events
RT: Radiotherapy
HR: Hazard ratio
VCA-IgA: IgA antibodies against Epstein–Barr virus capsid antigens
EA-IgA: IgA antibodies against the early antigen of Epstein-Barr virus
EBV: Epstein–Barr virus
